# MiR-1225-5p acts as tumor suppressor in glioblastoma via targeting *FNDC3B*

**DOI:** 10.1515/med-2020-0156

**Published:** 2020-09-09

**Authors:** Guo-Hua Wang, Liang-Yan Wang, Cui Zhang, Peng Zhang, Chuan-Hui Wang, Shuai Cheng

**Affiliations:** Department of Neurosurgery, Sunshine Union Hospital of Shandong Province, No. 9000 Yingqian Street, Weifang, Shandong, 261000, People's Republic of China; Department of Ophthalmology, The Affiliated Hospital of Weifang Medical University, Weifang, Shandong , 261000, People's Republic of China

**Keywords:** glioblastoma, miR-1225-5p, FNDC3B, target, regulate

## Abstract

This study attempted to research the molecular mechanism underlying the inhibitory role of miR-1225-5p in the malignant progression of glioblastoma. Bioinformatics analyses based on the gene expression omnibus (GEO) and Chinese glioma genome atlas (CGGA) databases showed that miR-1225-5p, as a favorable prognostic factor, was expressed at low levels in glioblastoma, and its expression was also related to WHO grade and age. The subsequent CCK-8 assay indicated that miR-1225-5p might prevent the malignant progression of glioblastoma, which was represented by that miR-1225-5p mimic reduced the viability of glioblastoma cells. Then, we predicted that *FNDC3B* might be a potential target gene of miR-1225-5p, and it was negatively correlated with the level of miR-1225-5p, which were confirmed by dual-luciferase reporter assay, qRT-PCR and western blot assays. Moreover, based on the analyses of the cancer genome atlas (TCGA), Oncomine and CGGA databases, *FNDC3B* was enriched in glioblastoma and high expression of *FNDC3B* led to poor prognosis. Finally, CCK8 and transwell experiments showed that the ability of miR-1225-5p to inhibit glioblastoma cell viability, invasion and migration was at least partially achieved by targeting *FNDC3B*. In general, these results revealed that the miR-1225-5p/*FNDC3B* axis contributes to inhibiting the malignant phenotype of glioblastoma cells, which lays a foundation for molecular diagnosis and treatment of glioblastoma.

## 1 Introduction

Glioma is one of the most malignant primary brain tumors in human beings, accounting for 40–50% of primary intracranial tumors [1,2,3]. Glioblastoma is the result of further deterioration of glioma, accounting for 46.6% of all gliomas [4]. Glioblastoma has the characteristics of strong invasiveness, high recurrence rate and poor prognosis [5,6]. So far, surgical resection as well as postoperative radiotherapy and chemotherapy are still the main clinical treatment options [7,8]. Although there are many novel therapies, including endocrine therapy, targeted therapy, immunotherapy and oncolytic virus therapy, the prognosis is still poor [9]. Hence, the utilize of molecular targeted therapy for glioblastoma has become a research hotspot, which is also a potential direction of glioblastoma treatment in the future.

In recent years, due to the continuous development of research on miRNAs, it has been found that miRNAs played an increasingly important role in multiple carcinogenesis [10,11]. Accumulating studies have confirmed that miRNA is closely related to a variety of biological functions of cells [12]. Its deletion or overexpression, to a certain extent, can play a regulatory role in the biological behavior of tumor cells [13,14]. Moreover, miRNA can complement and combine with 3′-untranslation region (3′-URT) of the corresponding target gene to affect the target gene expression [15]. For example, miR-758-5p inhibits the malignant progression of glioblastoma via targeting ZBTB20 [16]. miR-198 can improve the chemosensitivity of temozolomide in glioblastoma through targeting O6-methylguanine-DNA methyltransferase (MGMT) [17]. Consequently, it is not surprising that a part of miRNAs contribute as tumor suppressors in glioblastoma. These miRNAs include miR-1225-5p, which has been reported to have anticancer impacts in a variety of cancers [18,19]. miR-1225-5p was downregulated in thyroid cancer, which inhibited the malignant phenotype of thyroid cancer cells by regulating SIRT3 [20]. It also plays an anticancer role in osteosarcoma, and this function was partially reversed by Sox9 [18]. Importantly, Li et al. found that miR-1225-5p involved in glioblastoma pathogenesis as a tumor suppressor [21]. Nevertheless, according to the previous studies, an miRNA may adjust hundreds of genes at posttranscriptional level, and a gene can be targeted by multiple miRNAs [22], and hence, its in-depth mechanism still needs further study.

In this article, bioinformatics prediction suggested that *FNDC3B* may be a target gene of miR-1225-5p, which was expressed at high levels and led to poor prognosis. Based on the literature and the bioinformatics analysis, we designed to affirm the function of miR-1225-5p in glioblastoma and explore whether this function is achieved by regulating *FNDC3B*, hoping to provide more extensive data support for the clinical application of miR-1225-5p in glioblastoma.

## 2 Materials and method

### 2.1 Data collection

The differential expression of miR-1225-5p in glioblastoma patients was analyzed based on the data from GEO database (access number: GSE93850 and GSE139031), which totally includes 192 glioma patients and 165 healthy control. The mRNA expression data of 168 glioblastoma patients and 110 healthy controls were downloaded from TCGA database to analyze the expression of *FNDC3B* in glioblastoma and control. Then, we further validated the expression of *FNDC3B* in the glioblastoma using the Oncomine database (Sun Brain dataset).

The clinical information of the glioblastoma patients, including WHO grade, age and overall survival (OS) time, were obtained from the CGGA website (microRNA_array_198, mRNAseq_325, mRNAseq_693, mRNA_array_301).

### 2.2 Cell culture and transfection

Human glioblastoma cell lines U87, U251 and A172 were obtained from American Type Culture Collection (ATCC, American). Normal control cell (normal human astrocytes, NHA) was purchased from Sciencell Research Laboratories (American). RPMI-1640 medium supplement with 10% FBS, 100 U/mL penicillin and 0.1 mg/mL streptomycin was used to routinely culture the cells at 37°C in 5% CO_2_.

The miR-1225-5p mimic/inhibitor and negative control were purchased from Shanghai GenePharma Co., Ltd (Shanghai, China), which were used to upregulate/downregulate the expression of miR-1225-5p. For overexpression/knockdown of *FNDC3B*, we also synthesized si-con (5′-CGAACUCACUGGUCUGACC-3′), si-*FNDC3B* (5′-GAUGGAAAUUCUGAAGCGAAUCAGT-3′), pcDNA3.1-*FNDC3B* and corresponding control vectors from Shanghai GenePharma Co., Ltd (Shanghai, China). Lipofectamine 2000 Reagent (Invitrogen, Carlsbad, CA, USA) was used for transfection, following the manufacturer’s protocol.

## 3 RNA extraction and qRT-PCR

The TRizol reagent (Thermo, Massachusetts, USA) was used to extract the total RNA of cells based on the manufacturer’s protocol. Then, reverse transcription of total RNA into cDNA was performed using M-MLV (Clontech, Palo Alto, USA) and mi Script PCR reverse transcription kit (Qiagen, Dusseldorf, Germany), according to the manufacturer’s instructions. Afterward, MiScript SYBR-Green PCR kit (Qiagen) and SYBR Premix Ex Taq II (TaKaRa, Japan) were used to detect the expression levels of miR-1225-5p and *FNDC3B*, respectively. *U6* and *GAPDH* were used as the internal reference genes. The qRT-PCR reaction was carried out as follows: 95°C, 5 min; 95°C, 5 s; 60°C, 30 s, 40 cycles. The 2^−ΔΔCt^ method was used to calculate the expression levels of miR-1225-5p and *FNDC3B*.

The specific primer sequences were as follows: miR-1225-5p: F: 5′-GGGTACGGCCCAGTG-3′ and R: 5′-GAACATGTCTGCGTATCTC-3′; U6: F: 5′-CTTGGCAGCACATATACT-3′ and R: 5′-AAAATATGGAACGCTTCACG-3′; *FNDC3B*: F: 5′-GAAAGTCTCCCTGTTCGCACAC-3′ and R: 5′-ACTCTGAGACCTCACAGCCACT-3′; GAPDH: F: 5′-CCACTCCTCCACCTTTGAC-3′ and R: 5′-ACCCTGTTGCTGTAGCCA-3′.

## 4 Western blotting assay

Lysis buffer was used to extract the protein from cells, followed by quantifying the protein content using a BCA protein quantitative kit. Subsequently, 10% SDS-PAGE was used to separate the proteins and then transferred the isolated protein sample to polyvinylidene fluoride (PDVF) membranes. Membranes were sealed with Western Sealing Solution (5% skimmed milk powder) for 1 h and then incubated overnight with primary antibodies (*FNDC3B*; [1:200, Abcam, Cambridge, UK] and GAPDH [1:10,000, Abcam, Cambridge, UK]) at 4°C. After washing, the membranes were incubated with the secondary antibody (Santa Cruz Biotechnology, Inc., USA) at room temperature for 1 h. Then, the images were developed with electrochemical luminescence (ECL, Thermo Fisher Scientific, Inc.). GAPDH was used as a control to evaluate the relative protein levels.

### 4.1 Bioinformatics prediction

Bioinformatics algorithm from miRDB (http://www.mirdb.org/) was used to predict the target genes of miR-1225-5p.

### 4.2 Dual-luciferase reporter assay

The wild-type (WT) 3′-untranslated region (UTR) and the Mutant (Mut) 3′-UTR of *FNDC3B* were cloned into pmiR-RB-REPORTTM dual-luciferase reporter assay carrier. These plasmids were co-transfected with miR-1225-5p mimic/inhibitor/NC into cells using Lipofectamine 3000, as directed by manufacturer. After transfection for 48 h, the cells were gathered and submitted for the luciferase analysis with the Dual Luciferase Reporter Assay Kit post (Promega Corporation, USA).

### 4.3 CCK-8 assay

The cultured cells were digested and then used to prepare cell suspension. Cells were seeded in 96-well plates at the density of 1,000 cells/well and routinely cultured in a carbon dioxide incubator. Cell viability was measured at 0, 24, 48 and 72 h. At the indicated time points, 10 μL of CCK-8 solution was added to each well and then incubated for 1.5 h at 37°C. The optical density (OD) was calculated by the microplate reader (Bio-Rad, CA, USA) at 450 nm wavelength. The multiplication curve was drawn up using the OD value.

### 4.4 Transwell assay

First, we precoated 40 μL of Matrigel Matrix glue (Sigma-Aldrich) in the upper chamber and incubated the chamber overnight at 37°C to make the Matrigel gelatinous. Migration assay did not require gluing treatment. After digestion, cells in serum-free culture medium (at a concentration of 1 × 10^5^/mL) were added to the upper chamber and 500 μL of complete culture medium was added to the lower chamber and then cultured the chamber at 37°C for 24 h in a 5% CO_2_ incubator. After incubation for 3 h (for migration) or 16 h (for invasion), the chamber was removed and the remaining cells in the upper chamber were erased with a cotton swab. The cells in the lower chamber were fixed with 4% paraformaldehyde for 15 min and stained with 0.1% crystal violet for 3 min, followed by photographing them. Finally, five visual fields were randomly selected under optical microscope to observe and count the cells.

### 4.5 Statistical analysis

Data in this study were analyzed by using statistical analysis software SPSS22.0, and Student’s *t*-test (two groups) or one-way ANOVA variance analysis with posttest of Dunnett (multiple groups) was used for comparison between groups. Kaplan–Meier survival analysis was used to plot the survival curves, and log-rank test was used for comparison. The high-expression group and the low-expression group were grouped based on the median expression levels of miR-1225-5p and *FNDC3B*. *P* < 0.05 indicated that the difference has statistical significance.

## 5 Results

**Figure 1:**
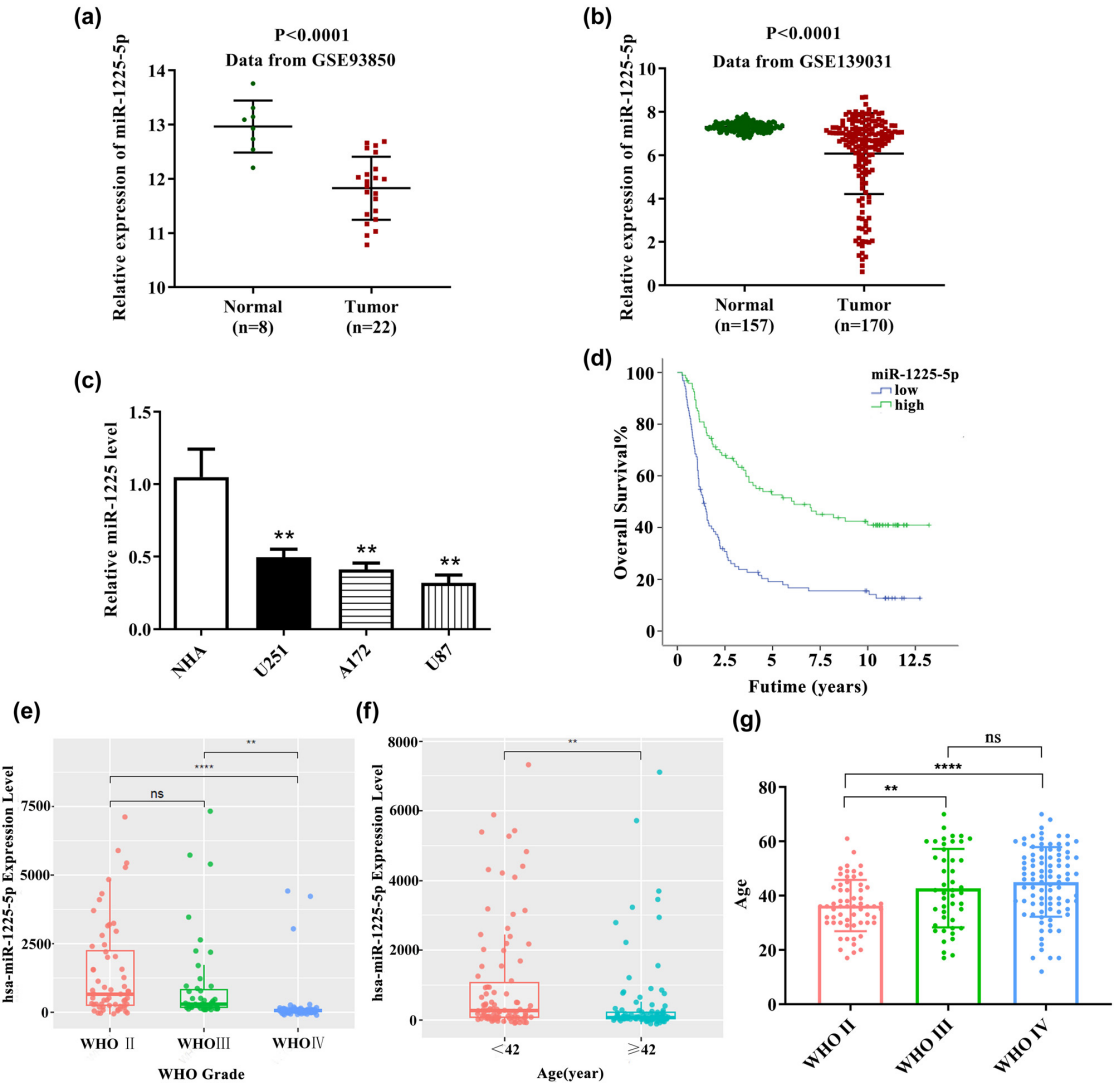
miR-1225-5p was expressed at a low level in glioblastoma and related to WHO grade, age and prognosis. (a) The differential expressions of miR-1225-5p in glioblastoma patients (*n* = 8) and healthy control (*n* = 22) were analyzed based on the GEO database (GSE93850). (b) The data containing 170 glioblastoma patients and 157 healthy controls were downloaded from GEO database (GSE139031) to analyze the differential expression of miR-1225-5p. (c) qRT-PCR was carried out to detect the expression of miR-1225-5p in glioblastoma cell lines (U87, U251and A172) and control (NHA). Then, (d) the relationship between the miR-1225-5p level (high ([>112.30425, *n* = 95] or low [<112.30425, *n* = 95]) and glioblastoma patient survival was also assessed based on TCGA database. miR-1225-5p expression in (e) WHO stage II–IV glioblastoma and (f) patients with the age >42 or <42 was analyzed based on CGGA database (microRNA_array_198). Moreover, (g) the relationship between the age and the WHO grade in glioblastoma was also analyzed.

miR-1225-5p was expressed at low levels in patients with glioblastoma and glioblastoma cell lines, which was related to WHO grade, age and prognosis. We downloaded the glioblastoma data from GEO database (access number: GSE93850 and GSE139031) and CGGA (microRNA_array_198) to analyze the expression pattern of miR-1225-5p in glioblastoma. Based on the GEO database (access number: GSE93850 and GSE139031), miR-1225-5p was clearly upregulated in glioblastoma than that in the healthy control (Figure 1a and b). Then, we confirmed the miR-1225-5p level in glioblastoma cell lines using qRT-PCR assay. As expected, miR-1225-5p was lowly expressed in glioblastoma cell lines U87, A172 and U251 compared with the normal healthy cell NHA (Figure 1c). Interestingly, miR-1225-5p shows different expressions in different cell lines, of which U251 cell has the highest expression and U87 has the lowest expression. Moreover, we easily observed that the high-expression group (>112.30425, *n* = 95) and the low-expression group (<112.30425, *n* = 95) displayed remarkable differences in the survival rate of glioblastoma patients: the prognosis of patients with high expression of miR-1225-5p was better than that of patients with low expression of miR-1225-5p, indicating that miR-1225-5p is a favorable factor for the prognosis (Figure 1d).

The analysis results of data from microRNA_array_198 data set showed that the miR-1225-5p expression was correlated with WHO grade of glioblastoma patients that the higher the grade, the lower the expression. The miR-1225-5p expression in WHO IV was lower than that in WHO II and WHO III (Figure 1e). Second, the expression of miR-1225-5p was related to age, which was lower in patients older than 42 years than that in patients younger than 42 years (Figure 1f). Interestingly, we revealed that the age of glioblastoma patients was correlated with WHO grade that the higher the grade, the older the patients (Figure 1g). All these findings prompted that miR-1225-5p expression is associated with glioblastoma malignancy.

**Figure 2:**
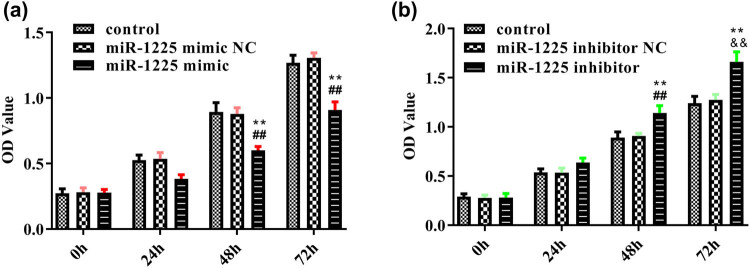
miR-1225-5p inhibits cell viability in glioblastoma. CCK-8 assay was performed to measure the effect of (a) miR-1225-5p mimic on U87 cell viability and (b) miR-1225-5p inhibitor in U251 cell viability. **P < 0.01 vs control group. ^##^ *P* < 0.01 vs miR-1225-5p mimic NC group. ^&&^ *P* < 0.01 vs miR-1225-5p inhibitor NC group.

miR-1225-5p inhibits cell viability in glioblastoma. First, for better verification of the function of miR-1225-5p in glioblastoma, based on the expression difference of miR-1225-5p in different cell lines, U87 cells were selected for overexpression experiments and U251 cells were selected for knockdown assays. Then, CCK8 assay was set up to assess the influence of miR-1225-5p expression on cell viability. The results of CCK8 experiment indicated that the upregulation of miR-1225-5p obviously declined the viability of U87 cells (Figure 2a), while inhibition of miR-1225-5p markedly increased the viability of U251 cells (Figure 2b). These results indicated that regulating the miR-1225-5p expression may alter the biological behaviors of glioblastoma cells.

**Figure 3:**
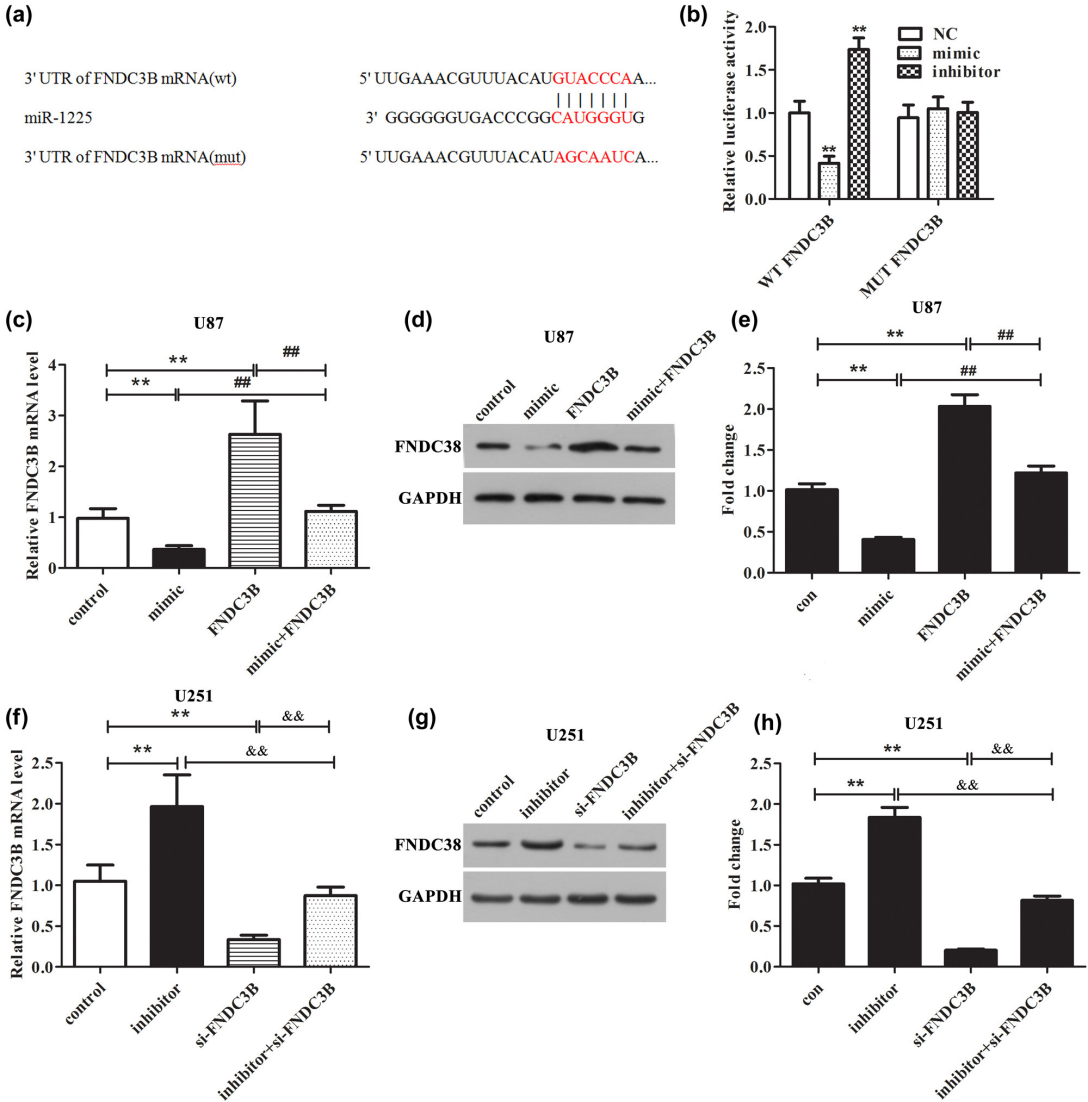
miR-1225-5p directly targets FNDC3B. (a) FNDC3B may be the potential target of miR-1225-5p, which was predicted by the miRDB website. (b) The luciferase activity assay was carried out to detect the luciferase activity of WT-FNDC3B and MUT-FNDC3B in cells transfected with miR-1225-5p mimic or inhibitor. Then, qRT-PCR and western blot were carried out to assess the mRNA and protein levels of FNDC3B in (c–e) U78 and (f–h) U251 cells. **P < 0.01 vs control group. ^##^ *P* < 0.01 vs miR-1225-5p mimic + pcDNA3.1-FNDC3B group. ^&&^ *P* < 0.01 vs miR-1225-5p inhibitor + si-FNDC3B group.

MiR-1225-5p was an upstream regulatory molecule of *FNDC3B*, which posttranscriptionally inhibited the expression of *FNDC3B* in glioblastoma cells. To identify the possible targets of miR-1225-5p participated in the progression of glioblastoma, target gene prediction software was used. The predicted results uncovered that miR-1225-5p might combine with the 3′-UTR of *FNDC3B* (Figure 3a). The luciferase activity assay was carried out to confirm whether miR-1225-5p directly targets *FNDC3B*. The results indicated that the upregulation of miR-1225-5p obviously declined the luciferase activity of the WT-*FNDC3B* 3′-UTR reporter, while inhibition of miR-1225-5p showed an opposite effect. However, miR-1225-5p-mediated changes in luciferase activity could be prevented by Mut binding sites (Figure 3b).

To further confirm the regulatory relationship between miR-1225-5p and *FNDC3B*, the mRNA and protein levels of *FNDC3B* were measured. From Figure 3c–e, we can easily observe that after overexpression of miR-1225-5p in U87 cells, both the mRNA and protein levels of *FNDC3B* were decreased significantly, while overexpression of *FNDC3B* can block the action of miR-1225-5p mimic in *FNDC3B* expression inhibition. Contrarily, the results shown in Figure 3f–h indicated that the inhibition of miR-1225-5p markedly increased the mRNA and protein expression levels of *FNDC3B* in U251 cell co-transfected with si-*FNDC3B* and miR-1225-5p inhibitor than that in miR-1293 inhibitor group. The aforementioned experimental results showed that miR-1225-5p directly targets *FNDC3B* and negatively regulates its expression.

**Figure 4:**
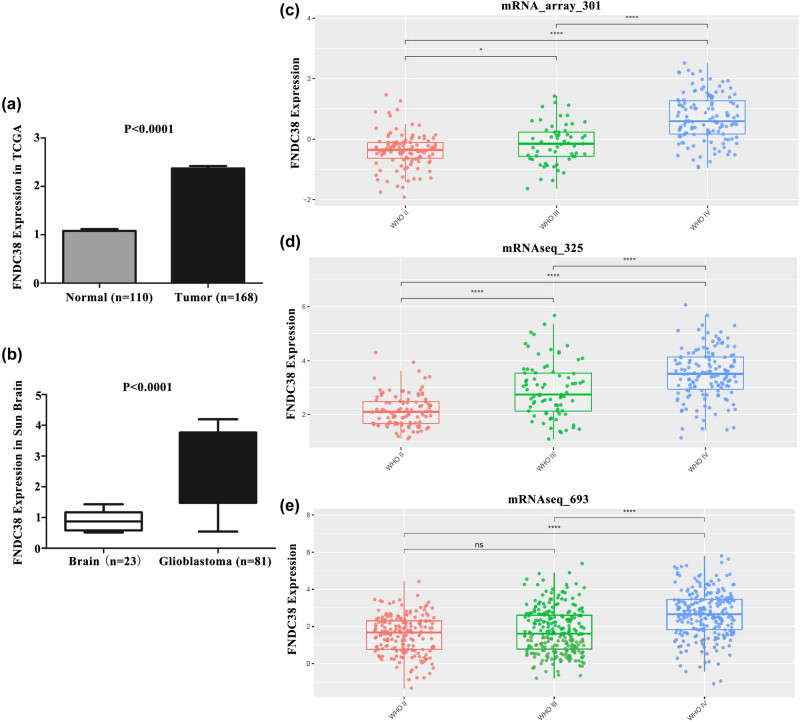
FNDC3B was highly expressed in glioblastoma. The differential expressions of FNDC3B in glioblastoma patients and healthy control were analyzed based on the (a) TCGA, (b) Oncomine Sun Brain, (c) CGGA mRNA_array_301 database, (d) CGGA mRNAseq_325 database and (e) CGGA mRNAseq_693 database.

*FNDC3B* was enriched in patients with glioblastoma and caused poor prognosis. The RNA-sequencing data of glioblastoma from TCGA, Oncomine and CGGA database were downloaded to evaluate the expression pattern of *FNDC3B*. As shown in Figure 4a–e, *FNDC3B* was strongly overexpressed in glioblastoma, which was related to the WHO grade of glioblastoma patients. WHO IV glioblastoma showed the highest *FNDC3B* expression compared with WHO II and WHO III glioblastoma, and the differences were statistically significant.

**Figure 5:**
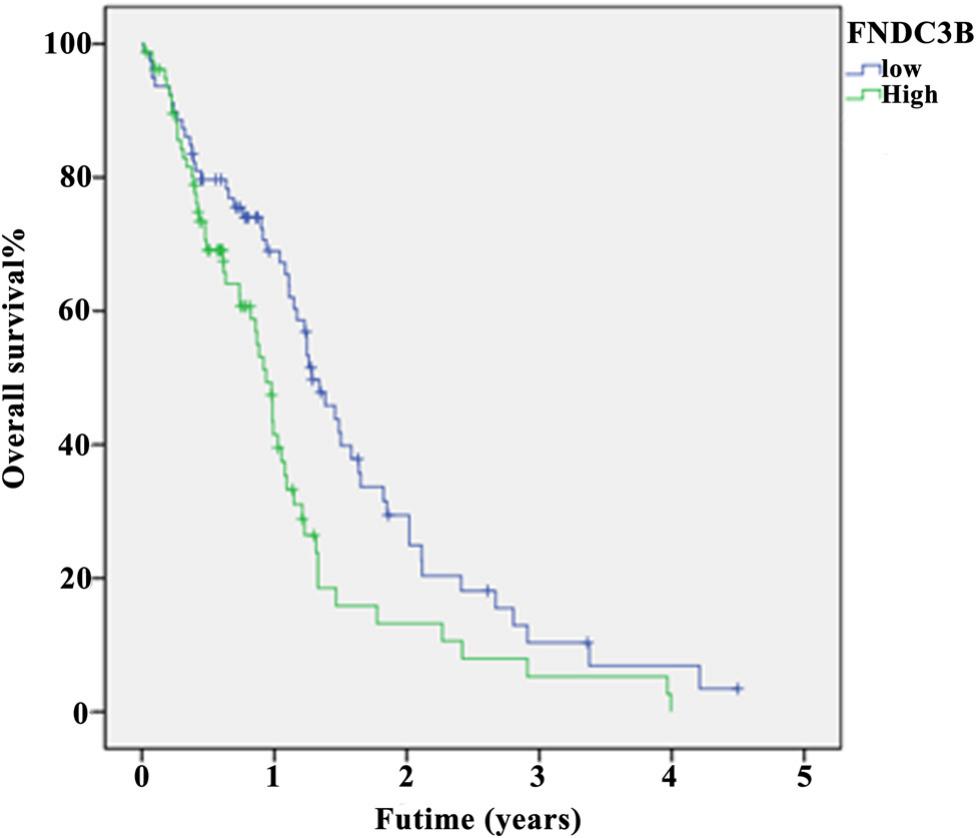
Overexpression of FNDC3B caused poor prognosis in glioblastoma. The relationship between the FNDC3B level (high [>1965.248214, *n* = 79] or low [<1965.248214, *n* = 79]) and glioblastoma patients’ survival was assessed based on TCGA database.

In addition, we divided into the high-expression group (>1965.248214, *n* = 79) and the low-expression group (<1965.248214, *n* = 79) based on the median level of *FNDC3B*. The prognosis analysis showed that the expression of *FNDC3B* was interrelated to the survival rate in glioblastoma patients, and unoptimistic prognosis was observed in patients with high expression (Figure 5).

**Figure 6:**
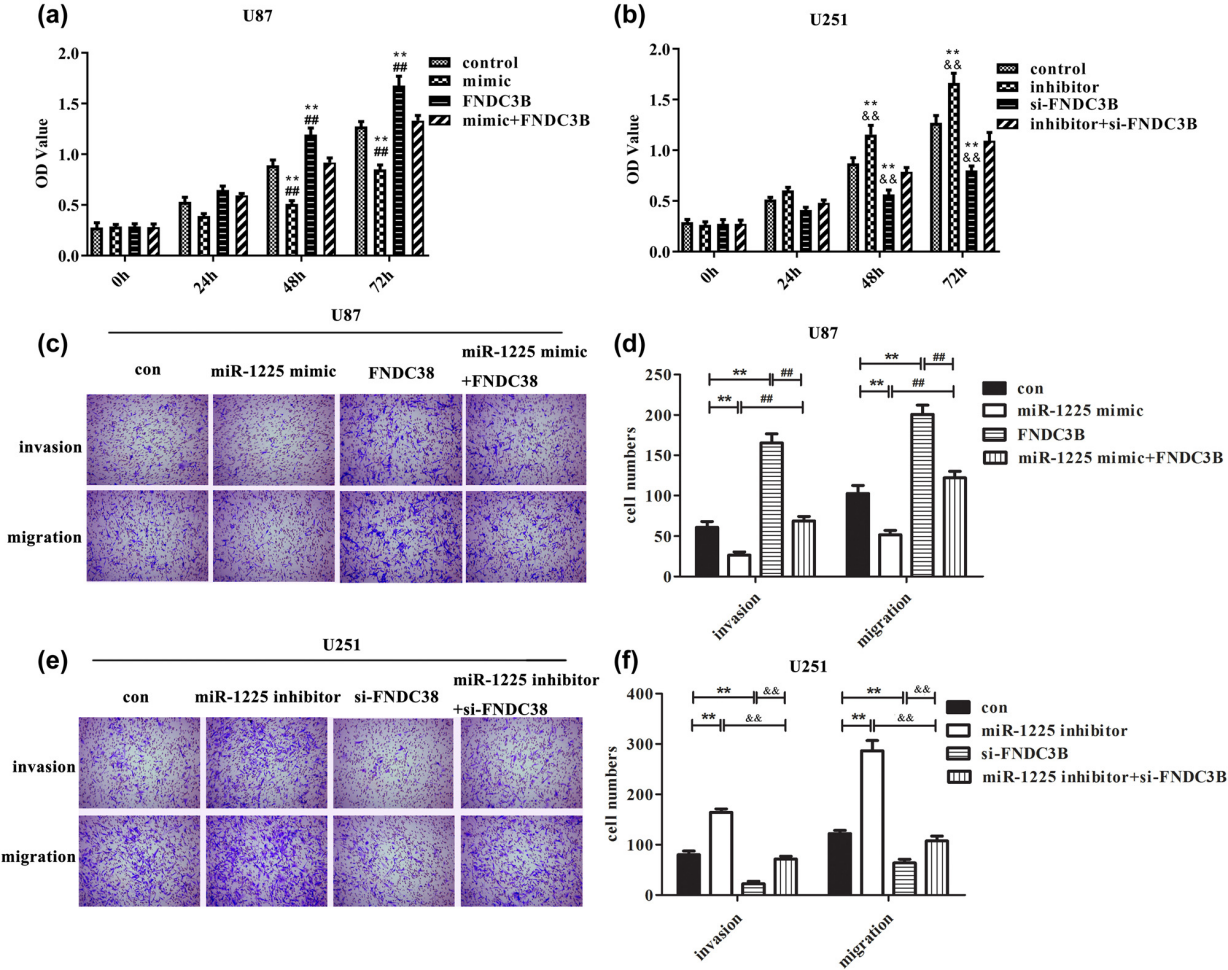
The effect of miR-1225-5p/FNDC3B axis on the malignant phenotype of glioblastoma cells. CCK-8 and Transwell assay were performed to measure the effect of miR-1225-5p/FNDC3B axis on cell viability, invasion and migration. (a, c and d) U87 cells were transfected with miR-1225 mimic, pcDNA3.1-FNDC3B or miR-1225 mimic + pcDNA3.1-FNDC3B. (b, e and f) U251 cells were transfected with miR-1225 inhibitor, si-FNDC3B or miR-1225 inhibitor + si-FNDC3B. **P < 0.01 vs control group. ^##^ *P* < 0.01 vs miR-1225-5p mimic + pcDNA3.1-FNDC3B group. ^&&^ *P* < 0.01 vs miR-1225-5p inhibitor + si-FNDC3B group.

Overexpression of *FNDC3B* prevents the miR-1225-5p-mediated suppression on malignant phenotype of glioblastoma cells. It has been reported that miR-1225-5p can inhibit cell viability and functioned as a tumor suppressor in glioblastoma [21], which was also verified by the aforementioned biological experiments. So as to explore whether this repressive function was realized by regulating *FNDC3B*, CCK-8 and transwell experiments were performed. As presented in Figure 6a and b, overexpression of *FNDC3B* showed an opposite effect of miR-1225-5p upregulation. Meanwhile, the cell viability was significantly increased when U87 cells co-transfected with miR-1255-5p mimic and pcDNA3.1-*FNDC3B* than that in miR-1255-5p overexpression group. On the contrary, knockdown of *FNDC3B* can reverse the influence of miR-1225-5p inhibitor on viability of U251 cells.

Furthermore, transwell assay results indicated that the number of cells invading and migrating was significantly reduced after U87 cells transfected with miR-1225 mimic compared with the control, and overexpression of *FNDC3B* has the opposite results. Simultaneously, it can be seen from co-transfection assay that upregulation of *FNDC3B* reversed the inhibitory effect of miR-1225-5p mimic on invasion and migration of U87 cells (Figure 6c and d). Conversely, inhibition of miR-1225-5p significantly promoted the migration and invasion of U251 cells, and knockdown of *FNDC3B* inhibited the effect of miR-1225-5p inhibitor on cell invasion and migration (Figure 6e and f). Taken together, these results illustrated that miR-1225-5p exerted the anticancer function in glioblastoma via regulating *FNDC3B*.

## 6 Discussion

Numerous research data showed that the high recurrence rate and mortality rate of glioblastoma are due to its uncontrollable excessive proliferation and high invasiveness of cancer cells [23,24]. In this study, we found that miR-1225-5p was expressed at low levels in glioblastoma, which is a favorable factor for the prognosis. Not surprisingly, the upregulation of miR-1225-5p restrained the viability of glioblastoma cells. Subsequently, for the first time, we determined that *FNDC3B* is a downstream target gene of miR-1225-5p, and miR-1225-5p negatively regulated its expression in glioblastoma. Furthermore, *FNDC3B* was highly expressed in glioblastoma, and its high expression was associated with unfavorable prognosis. More importantly, we affirmed that miR-1225-5p can inhibit the malignant phenotype of glioblastoma cells by regulating *FNDC3B*, including excessive cell proliferation, migration and invasion, which deepens our understanding of the mechanism underlying the involvement of miR-1225-5p in glioblastoma and the pathological mechanism of this disease.

As a member of miRNAs, the action of miR-1225-5p in cancer has been widely reported, including its antitumor effect in glioblastoma [21]. Our data confirmed that miR-1225-5p was expressed at low levels and inhibited the cell viability in glioblastoma, which is consistent with the conclusion that miR-1225-5p is a tumor suppressor of glioblastoma. Importantly, we also found that miR-1225-5p expression was related to WHO grade, age and prognosis. According to the classification of central nervous system tumors by the World Health Organization in 2016, gliomas are classified as WHO I–IV [25]. Our results indicated that the higher the WHO grade, the higher the age of patients and the lower the expression of miR-1225-5p. Also, our data revealed that the lower expression of miR-1225-5p indicated the shorter survival time of patients. Recently, it has been found that in primary spinal astrocytoma (a subtype of gliomas), the most significant factors associated with poor prognosis are the lesions with older age and higher histological grade at the initial diagnosis [26]. On the basis of references and the results of this study, we considered that miR-1225-5p is a biomarker for diagnosis and treatment of glioblastoma.

*FNDC3B* is mainly composed of fibronectin III (FNIII) domain [27], which has been widely reported to be upregulated in cancer as a oncogene [28], such as hepatocellular carcinoma [27], oral tongue squamous cell carcinoma [29] and gastric cancer [30]. This may be due to that the domain of FNIII was involved in cell adhesion [31,32], which can promote cell proliferation and migration, especially in the development of tumor [33]. Also, the characterization of *FNDC3B* in glioblastoma has also been studied. Stangeland et al. found that *FNDC3B* was expressed at high levels in glioblastoma [34], and our data confirmed this view. In addition, we demonstrated that patients with WHO IV glioblastoma showed the highest *FNDC3B* expression compared with WHO II and WHO III glioblastoma, and the higher the expression, the lower the survival rate. This suggested that *FNDC3B* may be a prognostic factor for glioblastoma. Since one miRNA may regulate hundreds of genes at posttranscriptional level and one gene can be targeted by many miRNAs, it forms a very complex regulatory network [22,35]. Herein, our study is the first to clarify the relationship between *FNDC3B* and miR-1225-5p in glioblastoma. We found that *FNDC3B* was the downstream target gene of miR-1225-5p, and based on the results of biological functional assays, overexpression of *FNDC3B* can reverse miR-1225-5p-mediated inhibition in glioblastoma. These findings indicated that miR-1225-5p/FNDC3B axis contributed to modulating the malignant progression of glioblastoma.

To sum up, our data concluded that miR-1225-5p is a favorable prognostic factor and tumor suppressor of glioblastoma, which can inhibit the proliferation, invasion and migration of glioblastoma cells by regulating FNDC3B. Our findings here prompted a novel light on the therapy of glioblastoma, but there are still some limitations in the current study. The findings of this study are restricted to glioblastoma cell lines *in vitro*, so the function of miR-1225-5p/FNDC3B axis *in vivo* needs further researches.
